# Erythrocytes as Potential Link between Diabetes and Alzheimer’s Disease

**DOI:** 10.3389/fnagi.2017.00276

**Published:** 2017-08-25

**Authors:** Cristiana Carelli-Alinovi, Francesco Misiti

**Affiliations:** ^1^School of Medicine, Biochemistry and Clinical Biochemistry Institute, Università Cattolica del Sacro Cuore Rome, Italy; ^2^Human, Social and Health Department, University of Cassino and Lazio Meridionale Cassino, Italy

**Keywords:** Alzheimer’s disease, diabetes mellitus type 2, red blood cells, amyloid beta peptide, oxidative stress, vascular disease

## Abstract

Many studies support the existence of an association between type 2 diabetes (T2DM) and Alzheimer’s disease (AD). In AD, in addition to brain, a number of peripheral tissues and cells are affected, including red blood cell (RBC) and because there are currently no reliable diagnostic biomarkers of AD in the blood, a gradually increasing attention has been given to the study of RBC’s alterations. Recently it has been evidenced in diabetes, RBC alterations superimposable to the ones occurring in AD RBC. Furthermore, growing evidence suggests that oxidative stress plays a pivotal role in the development of RBC’s alterations and vice versa. Once again this represents a further evidence of a shared pathway between AD and T2DM. The present review summarizes the two disorders, highlighting the role of RBC in the postulated common biochemical links, and suggests RBC as a possible target for clinical trials.

## Introduction

Patients affected by Alzheimer’s disease (AD) have senile plaques in central nervous system (CNS) areas where neurodegenerative process takes place (Selkoe, 1994). AD plaques are composed principally by amyloid β-peptide (Aβ), that can be formed by 39–43 amino acids and that derives from a longer precursor (APP) localized in transmembrane. Aβ, as showed by Yankner et al. (1990), is neurotoxic especially in aggregate form and it can lead to apoptosis of neuronal cells.

Although, historically, amyloid plaques were thought to cause AD (Hardy and Higgins, 1992), recent data suggest that Aβ oligomers, instead of plaques, trigger the pathological process (Roychaudhuri et al., 2009). On the basis, many studies have investigated the patho-mechanisms and to identify the risk factors of the disease. Vascular related diseases such as diabetes, hypertension and hypercholesterolemia have been reported to favor AD (Helzner et al., 2009) and in addition, AD patients have an increased risk of stroke events (Chi et al., 2013; Tolppanen et al., 2013). These data suggest a reciprocal relationship between vascular risk factors and AD.

It has been shown that Aβ causes oxidative stress (Nunomura et al., 2001). Beside its presence in CNS, Aβ can be detected in platelets (Chen et al., 1995) and blood (Seubert et al., 1992), where it interacts with red blood cells (RBC). Our previous studies (Clementi et al., 2004; Misiti et al., 2012; Carelli-Alinovi et al., 2015a, 2016a) suggest that Aβ is able to alter RBC metabolism. Other studies (Jayakumar et al., 2003; Mandal et al., 2003; Nakagawa et al., 2011; Lang and Lang, 2015), indicate that Aβ could impairs RBC functionality and integrity, enhancing abnormalities at the vascular level that could be responsible for AD (de la Torre, 2002). Aβ induces oxidative injury to RBC (Kay, 1984; Mandal et al., 2003; Clementi et al., 2007) that can lead to eryptosis (Nicolay et al., 2007). Diabetes is accompanied with impaired microcirculation resulting in relative tissue hypoxia (Ditzel and Standl, 1975; Ditzel et al., 1978). It induces numerous abnormalities in RBC, responsible for microcirculation impairment (Le Devehat et al., 1994), including increased aggregation (Schmid-Schönbein and Volger, 1976) and membrane viscosity (Baba et al., 1979) and decreased deformability (McMillan, 1975; Vague and Juhan, 1983). Reduced viscoelastic properties of RBC membranes have been attributed to the alterations in membrane lipid-protein interactions. RBC membrane is altered by free radicals-induced oxidative stress (Kumar, 2012). Diabetic altered RBC became able to adhere to endothelium by a specific interaction between advanced glycation end products (AGE) present on RBC and a specific receptor on the endothelium (Grossin et al., 2009). Once again, all this morphological, structural and biochemical changes result in an accelerated clearance of RBC. In the present review, we describe RBC main alterations in a diabetic milieu and in AD and refer to common pathophysiological mechanisms linking these two diseases.

## Evidences for A Connection Between Type 2 Diabetes and Alzheimer’s Disease

Previous studies (Ott et al., 1999) show that AD is more frequent in type 2 diabetes (T2DM) patients. These findings indicate that glucose dysmetabolism is involved in AD onset. Glucose dysmetabolism occurs in brain regions during pre-symptomatic period, although further studies will be needed to clarify if glucose dysregulation starts AD pathology, or it is a secondary effect due to Aβ-related toxic events and tau formation (Sato and Morishita, 2015). Recent studies demonstrate that individuals with T2DM develop AD with high frequency (Ott et al., 1999; Crane et al., 2013; Huang et al., 2014), and patients with hyperglycemia are more prone to develop AD from mild cognitive impairment (MDI; Morris et al., 2014). In addition, T2DM-related conditions, including obesity (Beydoun et al., 2008), hyperinsulinemia (Peila et al., 2004) and metabolic syndrome, may be risk factors for AD.

In this regard, there are in literature data on impaired insulin action or production, impaired signaling pathway involving insulin receptor (IR) and insulin growth factor (IGF) defects, toxicity caused by hyperglycemia, increase of advance glycation end products, inflammation at the vascular level and others (Sjöholm and Nyström, 2006; Luchsinger, 2012; de la Monte, 2012). It has been found a reduction in neuronal insulin transport, uptake and concentration in animal studies (Baskin et al., 1985; Banks et al., 1997; Kaiyala et al., 2000).

Recently Takeda et al. (2010) reported that defects in insulin-like growth factor 1 (IGF-1) receptor, IR and insulin receptor substrate (IRS)-1/2, suggesting that these events could be involved in the mechanism underlying AD and diabetes. Insulin signaling impairment leads to loss of neuronal function, plaque formation and NTF formation (Biessels and Kappelle, 2005). Aβ is able to bind IR in a competitive way, inhibiting its auto-phosphorylation and downstream kinases necessary for neuronal function in hippocampal region (Townsend et al., 2007). It was shown that mice in hyperglycemic or hyperinsulinemic status have a higher ability to generate Aβ in brain (Ho et al., 2004; Cao et al., 2007). Moreover Zhang et al. (2012) showed that in APP/PS1 mice model, Aβ correlate with insulin resistance and in humans with hyper-glycemia. In particular, in hepatocytes, they showed that Aβ induces insulin resistance, triggering JAK2/STAT3/SOCS-1 signaling pathway. Furthermore, insulin could interfere with the proteolytic Aβ degradation, that is known to occur via a metalloprotease, that recognize as substrates, also insulin and IGF-1 (Gasparini et al., 2002; Carro and Torres-Aleman, 2004; Plum et al., 2005; Carro et al., 2006; Moloney et al., 2010). High plasma insulin levels, occurring in insulin resistance patients, may be responsible for insulin-degrading enzyme inhibition; this event, as reported by the authors, impairs Aβ degradation, favoring its toxicity (Gasparini et al., 2002; Carro and Torres-Aleman, 2004; Plum et al., 2005; Carro et al., 2006; Moloney et al., 2010). Insulin resistance promote tau phosphorylation, leading to glycogen synthase kinase-3β activation (Li et al., 2006; Kremer et al., 2011). Genetic factors are involved in diabetes and AD cognitive impairment such as apolipoprotein E (ApoE). For example, ApoEε4 allele is present in the “late onset familial” and the “sporadic” ones, both forms of AD (Corder et al., 1993). A previous study has shown synergistic effects between the ApoEε4 and diabetes for developing AD (Peila et al., 2002).

AD is promoted by a T2DM status and their linkage is furthermore influenced by many factors, including ethnicity, glycemia and insulin. Thus, it becomes important to understand which of these factors are more decisive in the correlation between AD and T2DM, considering that in literature there are several controversial data (Neuropathology Group of the Medical Research Council Cognitive Function and Ageing Study (MRC CFAS), 2001; Petrovitch et al., 2001; Peila et al., 2002). In particular, Alzheimer-type pathology was found less frequent in diabetic patients when compared to non-diabetic subjects (Nelson et al., 2009).

## Red Blood Cells in Type 2 Diabetes

Diabetes is characterized by microvascular alteration (Jones and Peterson, 1981) and in diabetic RBC have been observed several functional and structural alterations (Jones and Peterson, 1981), such as a reduced life span (Peterson et al., 1977; Pescarmona et al., 1982), excessive aggregation (Schmid-Schönbein and Volger, 1976; Satoh et al., 1984), altered membrane phospholipid asymmetry (Wali et al., 1988), and a higher tendency to adhere to endothelial cells (Wautier et al., 1981; Wali et al., 1988). RBC alterations are linked to glucose metabolic disorder, whereas, others are associated with diabetes-related dysfunctional mechanisms (Jain et al., 1983). The main important mechanisms that affect RBC structure and function in diabetes patients are given below.

### Oxidative Stress

RBC compared to other cells are more affected by oxidative damage occurring in diabetes (Beisswenger et al., 2005), because of its higher levels of iron and poly-unsatured fatty acids. High blood glucose concentration causes phosphatidylserine (PS) exposure, a marker of eryptosis, triggering RBC removal by macrophages (Boas et al., 1998; Eda and Sherman, 2002). Similarly, methylglyoxal inhibits ATP production and decreases GSH levels (Nicolay et al., 2006), resulting in PS exposure, and eventually in anemia and microcirculatory disequilibrium (Nicolay et al., 2006).

### Lipids and its Modifications

Fatty acid composition changes in T2DM RBC. In particular, arachidonic acid and the total content of n-6 fatty acids were inversely proportional to the plasma insulin content during in fasting conditions (Clifton and Nestel, 1998). In diabetic patients, the saturated fatty acid amount was higher than in control and at the same time polyunsaturated fatty acid levels were lower than control (Prisco et al., 1989). Moreover, it has been evidenced a decrease in cholesterol/phospholipids ratio (Maksina et al., 1992; Mawatari et al., 2004) with a concomitant increase in sphingomyelin/phosphatidylcholine ratio. This situation may cause, at least in part, RBC function abnormalities and insulin resistance, because of inconvenient membrane fluidity. Previous studies have reported in plasma of diabetic patients and rats, high levels of oxidized lipids-derived aldehydes (Sato et al., 1979; Matkovics et al., 1982; Kaji et al., 1985; Uzel et al., 1987; Dohi et al., 1988). Further *in vitro* studies have reported that, damaging effects of hydrogen peroxide on RBC of diabetic patients is more relevant with respect to control ones (Matkovics et al., 1982; Uzel et al., 1987). Oxidized lipids, such as TBARS and conjugated dienes localized in RBC membranes, favor vascular defects reported in diabetes patients (Baynes, 1991; Jain et al., 1989). It is not yet known the mechanism responsible for hyperglycemia-induced membrane lipid peroxidation. It has been also reported that lipid peroxidation levels are correlated with the levels of HbA1c (Jain et al., 1989), as well as the 7-oxocholesterol/cholesterol and conjugated linoleic acid/linoleic acid ratios (Inouye et al., 1998, 1999). Some articles report that glucose reduces oxygen, leading to formation of aldehydes, H_2_O_2_ and ROS, resulting in oxidative stress and MDA (Carrell et al., 1975; Ramasarma, 1982; Halliwell and Gutteridge, 1984).

### Protein Modifications

AGE (Basta, 2008) found in high levels in diabetic RBC affect cell survival, affecting protein integrity involved in membrane structure (Elkrief et al., 2016). Previous evidences show that increased blood glucose levels cause RBC membrane protein glycation, resulting in higher cell fragility (Hatanaka et al., 2016). Higher glycosylation levels affect cytoskeleton, resulting (Cho et al., 2008) in morphologically abnormal RBC with decreased life span (Labrouche et al., 1996). Serum protein glycation causes modification in RBC proteins and modulates their biological or structural function (Cho et al., 2008). Among RBC membrane proteins, the glucose transporter-1 (Glut-1) is responsible for basal glucose uptake and transport across the plasma membrane (Kawano et al., 1999). Several evidences showed that Glut-1 is more susceptible for glycation and its increased structural alteration in diabetes causes cellular and tissue damage (Bonadonna et al., 1996).

### Adhesion to Endothelium

Adhesion of diabetic RBC to endothelium is mediated by a specific interaction between AGE, present on the RBC and a specific receptor on the endothelium. Consistent with this hypothesis, diabetic RBC of rats were able to interact with blood vessels receptor for advanced glycation end products (RAGE), and this event was followed by oxidative stress generation (Wautier et al., 1994). Furthermore, consequence of the RBC/endothelium interaction results in several perturbations such as an increased vessel permeability and interleukin-6 (IL-6) production. The oxidant stress secondary to RBC adhesion induces the activation of NFκB. The presence of RAGE in different cell types suggests that it could be involved in the genesis of diabetic complications, although the exact involvement of the AGE–RAGE interaction needs additional evidence. Inhibition of nitric oxide (NO) formation by nitro-L-arginine, potentiates the adhesion of RBC from diabetic patients to endothelium (Grossin et al., 2009). By contrast, the addition of NO donors (NOR-3, SIN-1 or SNAP) reduced or inhibited adhesion of RBC from diabetic patients measured in flow conditions (Grossin et al., 2009).

### Alterations in RBC Deformability

Alterations in membrane lipid asymmetry and composition, as well as cytoskeletal ones, alter RBC shape and deformability, subsequently responsible for reduced RBC membrane integrity, when encountering shear stresses (Bennett-Guerrero et al., 2007). Structural alterations in RBC are reflected to the functionality of the cell. Previous data, supported by SEM visual analysis, have reported that RBC from diabetic patients differ in shape and size from RBC of healthy subjects (Buys et al., 2013). In addition, AFM analysis showed that T2DM RBC showed several structural and morphological alterations (Buys et al., 2013). Previously it has been observed an alteration in T2DM RBC ultrastructure, probably due to iron overcharge, that caused the polymerization of fibrinogen (Lipinski and Pretorius, 2013).

### Anemia

Low hemoglobin (Hb) levels is an indication of anemia and has the effect of inhibiting RBC from transporting oxygen to different tissues.

Anemia is easily found in people with diabetes and contributes to the pathogenesis of complications related to diabetes (Astor et al., 2002), especially in cases of renal dysfunction (Dikow et al., 2001; Herzog et al., 2008). A positive correlation was found between anemia, retinopathy (Qiao et al., 1997) and somatic neuropathy in T2DM patients (Mezzano et al., 2003; Thomas et al., 2004; Herzog et al., 2008).

Chung et al. (2017) have demonstrated a correlation with cardiovascular autonomic neuropathy, supporting the hypothesis that anemia triggers neuronal injury (Mezzano et al., 2003; Thomas et al., 2004; Herzog et al., 2008).

The responsible mechanism is still not well understood, but bilirubin originated mainly by heme degradation (Berk et al., 1969), could play a central role due to its antioxidant mediated effects (Stocker et al., 1987; Kapitulnik, 2004).

Moreover, hyperglycemia is linked with an over-expression of proinflammatory cytokines (IL-6, TNF-α and NFkB; Martínez-Pérez et al., 2013; Angelousi and Larger, 2015) that lead to diabetic cardiovascolar complication and anemia (Barbieri et al., 2015). In particular IL-6 has an antierythropoietic effect promoting immature RBC apoptosis, ultimately responsible for Hb reduction (Fava et al., 2001; Angelousi and Larger, 2015).

## Altered RBC Functions in Alzheimer’s Disease

In last years, Aβ was found in peripheral plasma blood (Scheuner et al., 1996; Mehta et al., 2000; van Oijen et al., 2006; Graff-Radford et al., 2007) where it interacts with RBC, leading to impairment of its function (Mattson et al., 1997; Clementi et al., 2007; Mohanty et al., 2008), suggesting that this event could promote AD.

In particular, in AD RBC, have also been described physical alterations in membrane proteins (Kay et al., 1994), in the Ca++ permeability (Engström et al., 1995), in the antioxidant enzyme activities and morphological perturbations (Delibas et al., 2002) including Aβ-induced RBC suicidal death (i.e., eryptosis; Lang and Lang, 2015). There have been reports of links between RBC and AD. Some of these are highlighted below.

### Vascular Alterations

There is increasing attention to the vascular dysfunction as a possible cause of AD (de la Torre, 2002). In AD RBC, it has been reported a decrease in surface area, an increase in cell volume and alteration in membrane composition, leading to deformability decrease (Rifkind et al., 2002). Our recent *in vitro* data show a correlation between membrane alteration, signaling pathways activation and reduced RBC function (Carelli-Alinovi et al., 2016a). In AD patients, altered values for blood viscosity, mean corpuscular cell volume and RBC aggregation have been reported (Chang et al., 2007). An additional factor that can influence the oxygen delivery function in AD patients is the ability of RBC to adhere to the vasculature, by a mechanism mediated by Aβ. This kind of interaction causes a decrease in cell survival and the generation of oxidative stress and inflammatory condition (Nakagawa et al., 2011). Thus, authors suggested that Aβ and RBC interaction is responsible for blood flow alteration particularly at the cerebral level with amyloidosis.

### Metabolic Disturbances

Factors other than 2,3-DPG may affect the hemoglobin affinity to oxygen (Samaja et al., 2003). However, the relationship is established between the RBC concentration of 2,3-DPG and tissue hypoxia under various conditions. Thus, all above observations support the hypothesis that the chronic enhancement in the rate of active transport in RBC from AD patients leading to the decrease in the concentrations of ATP (Kosenko et al., 2012) and 2,3-DPG (Kaminsky et al., 2013), can result in increased hemoglobin affinity for oxygen, leading to impaired oxygen delivery to tissues (Aliev et al., 2009), which results in cognitive decline. In patients with AD, characteristic reduction of cerebral perfusion and metabolism occurs (de la Torre, 2002; Aliev et al., 2011) which inhibit the optimal delivery of glucose and oxygen (de la Torre and Aliev, 2005). The dysregulation of neuronal glucose metabolism in AD may result in a decrease in enzymatic activities of hexokinase (EC 2.7.1.1; Marcus and Freedman, 1997), phosphofructokinase (EC 2.7.1.11; Meier-Ruge et al., 1984), pyruvate dehydrogenase (EC 1.2.4.1) and enzymes of the tricarboxylic acid cycle (Bubber et al., 2005), and various other effects including the desensitization of the neuronal IR (Hoyer, 2000), impaired glucose transporter at the blood-brain barrier (BBB; Kalaria and Harik, 1989). At this regard, recently Kosenko et al. (2014) measured some parameters of adenine nucleotide metabolism, glycolysis, pentose phosphate pathway and the 2,3-DPG shunt in RBC from AD and age matched and young controls. From these results, it is clear that intracellular ATP levels, total adenine nucleotide pool size, and the ATP/ADP ratio were similar in RBC from AD patients and age-matched controls and lower than in young controls (Kosenko et al., 2012). However, activities of most of the enzymes such as hexokinase, glucose-6-phosphate isomerase (EC 5.3.1.9), phosphofructokinase, glyceraldehyde-3-phosphate dehydrogenase (EC 1.2.1.12), phosphoglycerate kinase (EC 2.7.2.3), pyruvate kinase (EC 2.7.1.40), lactate dehydrogenase (EC 1.1.1.27), glucose-6-phosphate dehydrogenase (EC 1.1.1.49), 6-phosphogluconate dehydrogenase (EC 1.1.1.44) and Na/K-ATPase, as well as the cytosolic NAD/NADH ratio, pyruvate and lactate levels, were higher in AD compared to controls, indicating an increase in RBC glycolysis and ion fluxes (Kaminsky et al., 2012, 2013).

### Protein Alterations

It has been shown that in late onset patients, RBC membrane glucose transporter protein 1 (GLUT1) and IR, as well as ATP-binding cassette transporter sub-family A member 1 (ABCA1) and ATP-binding cassette sub-family G member 2 (ABCG2), have higher levels of expression (Várady et al., 2015). For what concerns the early onset AD, it has been reported the same behavior outlined in the late-onset form for GLUT1 and IR, on the other hand no changes have been observed for RBC ABCA1, ABCG2, plasma-membrane Ca(2+)-ATPase (PMCA) and ATP binding cassette subfamily B member 6 (ABCB6; Várady et al., 2015). Generally, GLUT1 expression is modulated by glucose, hypoxia, insulin and growth hormones (Guo et al., 2005; Chen et al., 2015), and it has been shown that RBC GLUT1 expression is modulated by plasma elevated glucose levels (Harik et al., 1991). In the cases examined, where an up-regulation of GLUT1 and INSR was observed, a systemic hyper-glycemia was not present. Based on the relevant literature (Querfurth and LaFerla, 2010; Huang and Mucke, 2012), it could be hypothesized that GLUT1 and INSR increased levels, originate from transporter upregulation in endothelial cells of blood brain barrier due to brain hypoxia. IR level increase, could be caused by the insulin resistance in CNS (Querfurth and LaFerla, 2010; Huang and Mucke, 2012). Protein Kinase C (PKC) undergoes alteration in brain (Pascale et al., 2007) and in peripheral tissues in AD (Govoni et al., 1993; Solerte et al., 1998). It plays a relevant role in AD physiopathology in brain, because it is involved in the transduction pathway and changes that occurs include, expression level and translocation (Pascale et al., 2007). The PKC role in RBC is similar to brain isoform. Band 3, a transmembrane protein in RBC, has the same role in brain and it becomes phosphorylated by PKC and it has the same alterations observed in AD brain and RBC. These modifications include an altered conformation recognized by antibodies, a decrease in anion transport and in 32P-phosphate labeling (Bosman et al., 1991; Kay and Goodman, 1997). It could be suggested that band 3 alterations could be linked to an altered PKC activity. Our previous studies have shown that activation of RBC PKC after Aβ exposition could play a key role in oxidative imbalance, occurring following Aβ exposure (Carelli-Alinovi et al., 2015a). RBC mechanical properties are regulated by RBC PKC isoforms, caspase 3 and NO produced within the cell (Misiti et al., 2008; Carelli-Alinovi et al., 2015b).

### Oxidative Stress

In RBC with high ROS level it is easy to find higher levels of oxidized hemoglobin (metHb) that is unable to carry oxygen. Following incubation of RBC with different aggregate forms of Aβ peptides and Cu2+ i.e., mainly protofibril, it has been found that Cu2+-increased RBC oxidative stress. Oxidative stress leads to Hb oxidation, to the onset of heme degradation products on RBC membrane and to reduced ability to deform its shape.

Recent experiments have shown that band 3 is degraded by caspase 3 in Aβ treated RBC (Clementi et al., 2007), leading to band 3/glycolytic enzymes interactions abrogation (Mandal et al., 2003). This leads to an alteration of RBC metabolism (Galtieri et al., 2002), implying that, AD RBC have an increased risk of oxidative damage. At this regard, caffeine, a largely known antioxidant, reduced Aβ-induced toxicity in RBC (Carelli-Alinovi et al., 2016b). Although still controversial, malondialdehyde (MDA) levels of RBC membrane increase in AD patients (Skoumalová and Hort, 2012). RBC abnormalities might indicate the progression of AD oxidative damage. AD RBC have a greater membrane instability when exposed to H_2_O_2,_ compared with controls cells (Gilca et al., 2014). SOD activity protects cell but above a certain threshold, SOD is no longer able to protect and, on the contrary exalts peroxidation (Michiels et al., 1994). Therefore, SOD contributes to cell damage, favoring nitro-tyrosine formation in proteins. Previous articles suggested SOD as a peripheral marker of AD, reporting an increase in SOD activity in AD patient’s RBC (Serra et al., 1994, 2001).

### Anemia

Many studies have been conducted in an attempt to demonstrate a correlation between anemia and the development of the AD, but the results have often been discordant (Beard et al., 1997; Atti et al., 2006).

Low Hb levels could be a marker for ischemia, hypoxia-associated changes in hypoxia inducible factor (HIF) and erythropoietin levels, as well as alteration in heme regulation. Shah et al. (2011) demonstrated that hemoglobin levels were linked with cognitive decline. Chronic kidney disease associated with low Hb levels, could cause cerebral hypoxia, through a mechanism involving HIF and erythropoietin (Nangaku et al., 2008). At this regard, it has been suggested that a reduction in brain erythropoietin receptors (Maiese et al., 2005; Hasselblatt et al., 2006; Assaraf et al., 2007) increase neuronal degeneration.

Moreover, low hemoglobin levels expose RBC to a greater fragility, leading to an overload of heme molecules to be processed by astroglia brain. Elevated circulating free heme is able to over stimulate the hemo-oxigenase-1 activity, responsible for an increased oxidative stress, observed especially in sub-clinical AD subjects (Hascalovici et al., 2009).

RBC containing HbF are less vulnerable to oxidant injury with respect to those containing HbA, than they produce less Hb and heme, both of the have toxic effects upon the vascular cells. Consequently, elevated HbF could decrease hypo-perfusion and inflammation at brain level, and might be a protective factor for AD (Fallahzadeh et al., 2009). It has been also reported that lower hemoglobin levels are correlated with cognitive impairment and AD (Faux et al., 2014).

### Long-Chain Omega-3 Polyunsaturated Fatty Acids (PUFA)

Low levels of docosahexaenoic acid (DHA) were measured in AD RBC membranes (Wang et al., 2008), in fact it has been found that, in rat models, PUFAs supplementation was able to ameliorate the cognitive deficit (Hashimoto et al., 2002, 2006, 2008). DHA can change membrane composition, because it acts on cholesterol, on fatty acid composition and enhances antioxidant defenses.

## Anti Diabetic Therapy and Alzheimer’s Disease

Recent reports show that some antidiabetic drugs are able to induce neuronal survival, leading to clinical improvement of memory and cognition in different clinical settings. Studies on the effects of insulin therapy on the cognitive functions of dementia patients are controversial, some of these suggest that insulin increases the risk of dementia in diabetes patients, on the other hand further studies indicate that insulin slow down the cognitive decline in AD patients (Morris and Burns, 2012). Other drugs in addition to insulin such as metformin (Gupta et al., 2011), peroxisome proliferator–activated receptor (PPARγ; Jahrling et al., 2014), and incretins (Drucker, 2001) investigated for T2DM might potentially be beneficial for Alzheimer’s patients as well.

### Anti-RBC Therapy and Alzheimer’s Disease

As previously reported, RBC play a key role in T2DM and AD-associated vascular complications by increasing oxidative stress and may therefore favor an increased risk of developing AD in T2DM patients. However, several studies showed effectiveness of some antioxidants, no data are yet available as to whether antioxidants protect against AD. Reasons for these results might include, in part, BBB permeability, inappropriate timing of administration, or suboptimal drug levels at the target site in the CNS (Rutten et al., 2002; Gilgun-Sherki et al., 2003).

## Conclusions and Future Directions

Taking into account the literature discussed in this review, a strong correlation came out between T2DM and AD. In particular, it has been reported that T2DM subjects are at an increased risk of developing AD. Although both disorders possess several overlapping features, RBC abnormalities are relevant events and, at the same time, it is a “good indicator” of what happens at vascular level. Examples of this innovative idea, derive from recent literature focused on the understanding of RBC abnormalities that involve the impairment of RBC morphology, deformability, and function leading to vascular dysfunctions. Furthermore, and because RBC are major blood sources of ROS, the impairment of RBC functionality is accompanied by oxidative stress-related events. On this basis, altered RBCs following oxidative stress could be considered as a probable marker linkage for AD and T2DM. It is reasonable to enunciate that the current knowledge on the involvement of altered RBC dynamics in the pathogenesis of both AD and T2DM are still at an elementary stage, but it probable that elevated oxidative stress in RBC at vascular level both in AD and T2DM patients could combine with other factors, responsible for an increased risk of developing AD in T2DM patients. Furthermore, high levels of arachidonic acid and low levels of docosapentaenoic acid levels in RBC were observed in subjects with high neocortical beta-amyloid load, a feature of preclinical AD (Goozee et al., 2017), suggesting that inflammation and oxidative stress are early features of preclinical AD.

In light of its significant advancement from both clinical research and therapeutic application perspectives, we look ahead major research efforts being drawn to this field and more approaches being formulated soon. We also presume that finally these findings will be translated into novel drugs and effective therapies against both AD and T2DM triggered by RBC dysfunctions. The understanding of the molecular basis of these pathologies in RBC has the advantage to design a non-invasive diagnostic method, compared to the currently available techniques. Furthermore, it has been suggested that some of the drug currently used for T2DM might potentially be beneficial for Alzheimer’s patients as well.

## Author Contributions

FM and CC-A: substantial contributions to the conception or design of the work; drafting the work or revising it critically for important intellectual content agreement to be accountable for all aspects of the work in ensuring that questions related to the accuracy or integrity of any part of the work are appropriately investigated and resolved; FM: final approval of the version to be published.

## Conflict of Interest Statement

The authors declare that the research was conducted in the absence of any commercial or financial relationships that could be construed as a potential conflict of interest.
